# Text Mining Analysis of Korean University Students’ Academic Coaching Intake Session Reports

**DOI:** 10.3390/ijerph19106208

**Published:** 2022-05-20

**Authors:** Ahram Lee, Soo Jeung Lee, Jee Young Lee, Eunjeong Rhee

**Affiliations:** 1 Department of Education, Sejong University, Seoul 05006, Korea; leeyalan@sejong.ac.kr (A.L.); soojlee@sejong.ac.kr (S.J.L.); 2 Department of English Language and Literature, Korea University, Seoul 02841, Korea; jeeyoung@korea.ac.kr; 3 University Policy Research Institute, Korea University, Seoul 02841, Korea

**Keywords:** academic coaching, intake session, text mining, university student

## Abstract

Academic coaching has been emphasized in Korean universities as an effective measure to assist students’ academic achievement and success. To better assess the needs of the students, the current study investigated academic coaching intake session reports archived at a Korean university from January 2017 to August 2021 and examined students’ descriptions of their academic concerns and barriers. The intake session reports were categorized according to (1) students’ affiliated department tracks, namely Humanities and Social Science (HSS) and Science, Technology, Engineering, and Math (STEM) tracks, and (2) the time the coaching sessions took place, i.e., before and after the outbreak of COVID-19. Text mining analysis was conducted to calculate the frequency of keywords, their degree of centrality, and the frequency of bigrams, or the sets of two adjacent words, for each category. Wordclouds and word networks were also visualized. The results indicated that the word study was dominant in both categories, reflecting the education culture in Korea. Similarities and differences between the two categories were also reported. Based on the results, practical implications for academic coaches, educators, and university administrators were proposed, and limitations were discussed.

## 1. Introduction

### 1.1. Background and Purpose of the Study

Universities are where students develop academically, as well as intra- and interpersonally, and mature into autonomous and responsible citizens. In order to better prepare students for the ever-changing modern society, universities are emphasizing the role of personalized learning to tailor the learning process for each student [1,2] and the development of generic skills that are applicable in various fields [3,4]. Recognizing the rapid changes in society and transition of the education paradigm, universities in Korea have strived for educational renovation [5]. Specifically, the Ministry of Education in Korea has endeavored to enhance the quality of university education since the late 2000s and implemented policies such as the Advancement of College Education (ACE) Project and the University Innovation Support Project. Accordingly, universities have reconstructed curricula to offer competitive majors [6] and developed new extracurricular programs to support underachieving students [7]. Aligned with such efforts to focus on students’ development of competencies, universities have also increased customized interventions to support students’ academic achievement and campus life satisfaction.

Among the various student support measures that universities provide, academic coaching has been identified as one of the effective individualized interventions. Academic coaching involves a partnership between a trained coach and a student, based on which the student is empowered to set his or her own goals and learn new skills to attain academic success [8]. Although academic coaching is being widely implemented in universities, the studies thereof have only recently begun to accumulate, mainly because the history of the coaching field itself is relatively short [9]. Additionally, previous coaching-related studies have mainly targeted students in elementary, middle, and high schools [10,11]; studies on university students considered a specific population of students, such as those who were academically at-risk [12], with disabilities [13], or on the autism spectrum [14]. However, since the major purpose of coaching is to promote self-directed learning and personal growth [15], as well as academic achievement and success [10], it can be applied to any student who wants to improve their performance. Hence, more studies on academic coaching for general university students should be conducted.

In order to understand the needs of students and to devise coaching approaches to benefit more students, it was essential to first understand the range of issues brought to academic coaching by university students. For this purpose, the current study examined the intake session reports of an academic coaching program accumulated in the database at a four-year university in Seoul, Korea from 2017 to 2021 to gain understanding of the academic challenges that university students dealt with through coaching. Specifically, text mining analysis was applied to conduct a descriptive and exploratory study identifying keywords and their relations from the reports to highlight the aspects that students most frequently addressed in their initial coaching sessions. The study first made comparisons of the data according to two academic tracks, namely Humanities and Social Science (HSS) and Science, Technology, Engineering and Mathematics (STEM), and examined coaching issues before and after COVID-19 in order to raise awareness of coaches, educators, and university administrators regarding the academic needs of students.

### 1.2. Literatue Review

Coaching is aimed at helping a coached student set appropriate goals based on self-awareness and find feasible ways to achieve them [16]. It is a process of maximizing the potential of the coached student [17], through which the coached student learns to take new actions autonomously [18]. It is a customized process that fosters growth and action in those who participate. More specifically, academic coaching is a responsive and supportive process through which a coach and a student engage in a partnership to promote the student’s academic achievement and success [10]. Academic coaching is different from domain-specific intervention, such as tutoring, in that it does not teach content materials but focuses on empowering an individual to identify and solve one’s own problems [16]. It has been found to enhance university students’ academic self-efficacy [19], increase metacognition [20], and improve their self-directed learning ability [21,22], as well as their self-understanding and ability to set goals for college life [21]. A recent study that examined the effects of an intervention that integrated coaching with mindfulness also found that the participants experienced improvement regarding self-regulation, emotion, and motivation [23]. As such, academic coaching could be an effective support for all students who want to improve their performance. It is a content-general approach promoting students’ personal growth [15] and academic achievement [10].

In order to devise specific coaching approaches for university students, issues related to their university life and academic concerns should be addressed. Most of all, students transitioning from secondary school to university need to adjust to the new environment, navigating through courses and getting used to campus life [24]. In a university setting, students are required to be more self-directed [25] and adaptive to an array of novice experiences, including meeting people from different backgrounds and making choices among countless opportunities and activities [26]. Other factors, such as social support and loneliness, were found to be related to university students’ academic persistence [27]. Moreover, the results of Allen et al. [28]’s study reported various factors, including academic performance, academic self-discipline, pre-college academic performance, and social connectedness, to be directly or indirectly associated with third-year college retention and transfer. In addition, students’ campus life and academic performance can be influenced by behavioral factors, such as increased use of or addiction to cell phones [29,30,31] and emotional factors such as depression [32].

Student’s campus life and academic success can also be influenced by the characteristics of their affiliated departments, namely Humanities and Social Science (HSS) and Science, Technology, Engineering and Mathematics (STEM). Recently, universities in Korea have directed their attention toward providing customized learning support for students from HSS and STEM tracks. Previous studies have found that students from these two tracks showed significant differences in various areas; for instance, there were differences in the effects of subject satisfaction and relationship satisfaction on job-seeking stress [33], factors related to e-learning [34], and the tendency of general education enrollment, academic competencies, and career adaptability [35] between students affiliated with the HSS track and STEM track. Considering the different characteristics of these two tracks, it was hypothesized that students in each track would have different types of academic concerns or expectations.

Moreover, the prolonged outbreak of the pandemic affected university students, causing increased level of stress, anxiety, depression, and even suicidal thoughts [36] which could have impacted their academic performances, requiring adjustment to a remote instructional approach [37]. The pandemic changed the paradigm of higher education and students’ learning experiences thereof, and examining the keywords addressed before and after the pandemic could provide insight into understanding the academic concerns of university students.

Even though making an exhaustive list of these challenges or solving all the problems faced by university students through coaching is impracticable, the factors examined by previous studies were possible topics that could be addressed in academic coaching sessions. Understanding students’ major issues could assist coaches in devising more effective coaching interventions and guide educators and university administrators in generating the necessary support measures for students. Thus, the current study examined the keywords reported by students in coaching intake sessions to investigate the issues most frequently addressed in coaching sessions.

The current study used text mining analysis to investigate the frequency and relations of keywords addressed in academic coaching intake session reports. Text mining is using a computer system to extract previously unknown information from vast written texts and making links to generate new information [38]. Text mining analysis has been used in various studies in the field of higher education to investigate students’ feedback [39], opinions in online platforms [40], or the syllabi of higher education institutions [41]. As a text mining approach allows unstructured text data to be changed into structured data for analysis, it provides quantitative understanding of the natural and authentic data. Additionally, the extracted information can be linked together to build new hypotheses that can lead to future studies for more explorations [38]. In order to investigate a large number of accumulated session reports of coaching intakes, and to identify the keywords and their relations to provide base line data, text mining was applied in the current study.

## 2. Materials and Methods

### 2.1. Dataset

The current study analyzed the archived intake session reports of an academic coaching program in a four-year university in Seoul, Korea. The participants of the coaching program were students from the same university who had voluntarily signed up to receive coaching service from trained coaches. Prior to participating in the program, students were offered a separate consent form for collecting and using their coaching-related data for research in general, excluding any personal information. Students were also informed that their refusal to consent to research would not influence their access to the coaching service and that they could withdraw their consent to research at any time. Students who consented were asked to directly enter their information, including gender, grade level, affiliated department, and prior experience of academic probation on a computer database system. Then, an intaker, a coach who had completed intake training, conducted an intake session for each student in which the student described his or her academic concerns and barriers, perceived cause of difficulty, and expectations about coaching. The intakers who conducted the intake sessions were trained to write a report in an objective manner using the in vivo expressions of the students as much as possible.

In the current study, intake session reports collected from January 2017 to August 2021 were used for analysis. During this time, 10 intakers took part in writing the intake session reports. Initially, there were 464 intake session reports, but a total of 383 reports, excluding 81 reports about graduate school students, were used for analysis because the present study focused on undergraduate students. The constitution of the data was as follows: 145 males (37.9%), 235 females (61.4%), 3 unanswered (0.8%); 210 from HSS (54.8%), 171 from STEM (44.6%), 2 unanswered (0.5%); 256 before COVID-19 from 2017 to 2019 (66.8%), and 127 after COVID-19 from 2020 to 2021 (33.2%).

Information on grade level was also collected: 74 freshmen (19.3%), 131 sophomores (34.2%), 107 juniors (27.9%), 65 seniors (17%), and 6 unanswered (1.6%). However, the grade level was not considered as a factor of comparison in the analysis because it did not accurately reflect the status of students. For instance, there were students who were still in their freshman year after completing three or more semesters because they did not register and complete the required courses; there were also students who signed up for coaching at the end of the school year before immediately moving onto the next grade level.

Additionally, 48 students (12.5%) had prior experience of academic probation, indicated by a semester GPA below 1.75 out of 4.5; among them, only 7 students applied for coaching because of receiving academic probation in the prior semester. Due to the small number of students, the experience of academic probation was not included as a factor of comparison in the analysis.

### 2.2. Ethical Concerns

The current study used archived data from a database and did not collect any new data. Thus, the Institutional Review Board of Sejong University, Seoul Korea approved of IRB exemption (SUIRB-HR-E-2021-005, 18 August 2021).

### 2.3. Analytic Procedure

Text mining analysis was conducted using *R* program to examine the text of intake session reports collected from university students who voluntarily applied for the academic coaching program. Text mining used natural language processing technology in order to extract information from the given text, from which values were generated and hidden relationships revealed [42]. The analysis was conducted in the following process: calculation of frequency of keywords, analysis of the degree of centrality of keywords, and examination of the bigram.

First, the frequency of keywords was calculated. Since the original dataset of intake session reports was written in Korean, the initial analysis was conducted in the Korean language in order to accurately analyze the data. First, the stopwords in Korea (e.g., postpositional particle, conjunctive particle) were deleted. After calculating the frequency of keywords in Korean, the identified keywords were then translated into English. In the process of translation, one-word keywords in Korean were translated into two- or more words in English (i.e., high school, (do) not know, or leave of absence, etc.). To reflect the accurate frequency of the keywords from the original text, these English translations were intentionally put into one word (i.e., HighSchool, NotKnow, and LeaveOfAbsence, etc.). When translating the keywords from Korean to English, extra attention was paid to avoid changing the actual meaning of the word or the context. In order to ensure accuracy, the original text and the translation process and results were reviewed by one of the coauthors, who received a doctoral degree in English linguistics. The frequency of keywords was calculated based on two categories: students’ affiliated departments and coaching issues before and after COVID-19. For each category, keywords that appeared more than 30 times in the calculation were used to generate wordclouds. There were 156 keywords in the HSS track, 143 keywords in the STEM track, 200 keywords before COVID-19 (2017–2019), and 84 keywords after COVID-19 (2020–2021) with frequencies of 30 or more. The list of words is provided in Appendix A (Table A1, Table A2, Table A3 and Table A4).

Second, the degree of centrality of the keywords was analyzed. The degree of centrality refers to the number of links between words in the text [43]. A word with a higher degree of centrality indicated its higher centrality in the word network. A word network was displayed as a figure for each category, constructed with the top 30 keywords that had the highest frequency.

Finally, each category was investigated for bigrams. A bigram is a sequence of two adjacent keywords. This study investigated the frequency of sets of two words appearing together and presented the top 20 most frequently appearing bigrams.

## 3. Results

### 3.1. Keyword Frequencies

#### 3.1.1. Affiliated Department Tracks

**Figure 1:**
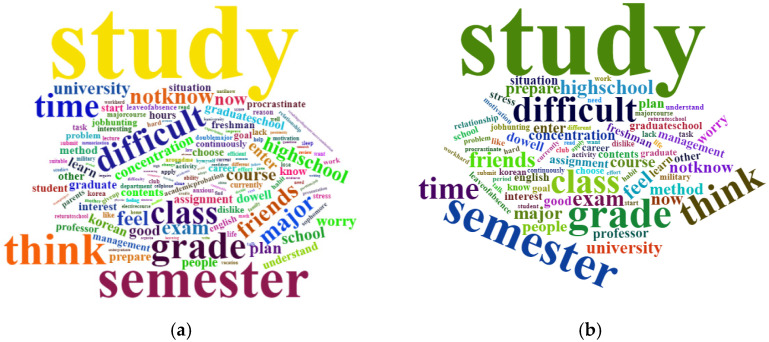
(**a**) Wordcloud of keywords for HSS track; (**b**) Wordcloud of keywords for STEM track.

There were 156 keywords with a frequency of 30 or more appearing 14,007 times in the HSS track, and 143 keywords appearing 11,760 times in STEM track. The word “study” was dominantly the most frequent word for both HSS track and STEM track, followed by “semester”. The next most frequent words showed similar patterns, albeit in different orders, for the tracks. For the HSS track, the next most frequent words were “think”, “grade”, and “difficult” in order of frequency; for the STEM track, these were “grade”, “think”, and “difficult”, respectively. The word “major” appeared more frequently in the HSS track while the word “exam” appeared more in the STEM track. Additionally, the words “plan”, “school”, “enter”, and “worry” appeared only in the HSS track, while “method”, “prepare”, “people”, and “concentration” appeared only in the STEM track—referring to words appearing among the top 20 keywords. The top 20 words are provided in Table 1, and the wordclouds are depicted in Figure 1.

#### 3.1.2. Coaching Issues before and after COVID-19

**Figure 2:**
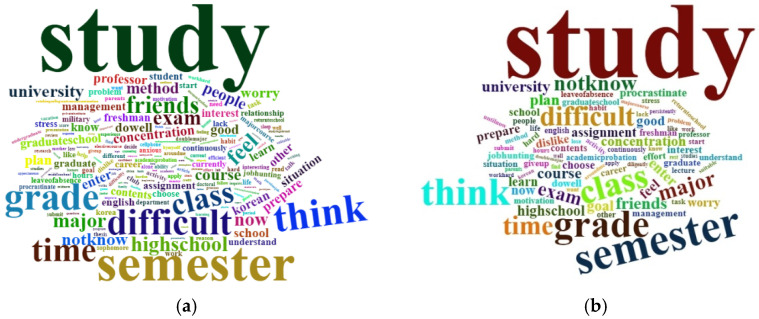
(**a**) Wordcloud of keywords before COVID-19; (**b**) Wordcloud of keywords after COVID-19.

There were 200 keywords with a frequency of 30 or more appearing 20,082 times before COVID-19 (2017–2019), and 84 keywords appearing 6166 times after COVID-19 (2020–2021). The word “study” was the most frequent word, followed by “semester”. Before COVID-19, words appeared in the following order: “think”, “grade”, “difficult”, and “time”. After COVID-19, the ranks of the words were as follows: “grade”, “think”, “class”, and “difficult”. Words such as “friends” and “feel” were relatively more frequent before COVID-19, while “major” and “NotKnow” were more frequent after COVID-19. Table 2 presents the top 20 words before and after COVID-19 and Figure 2 illustrates the wordclouds.

### 3.2. Centrality

#### 3.2.1. Affiliated Department Tracks

Centrality indicated a link with other words in the text; the higher the centrality, the more links there were. The word “study” showed the highest degree of centrality in both the HSS and STEM tracks, followed by “think”, “semester”, “class”, “difficult”, “grade”, and “time”. Table 3 presents the top 20 words and their degree of centrality.

**Figure 3:**
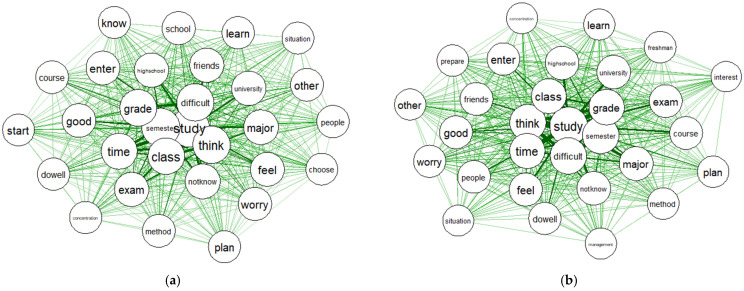
(**a**) Centrality of the top 30 keywords for the HSS track; (**b**) Centrality of the top 30 keywords for the STEM track.

Figure 3 presents the centrality of keywords, showing the links between the words. A thicker link indicates a higher degree of centrality.

#### 3.2.2. Coaching Issues before and after COVID-19

The word “study” showed the highest degree of centrality both before and after COVID-19, followed by “think” and “semester”. The centrality of the top ranked words is shown in Table 4.

**Figure 4:**
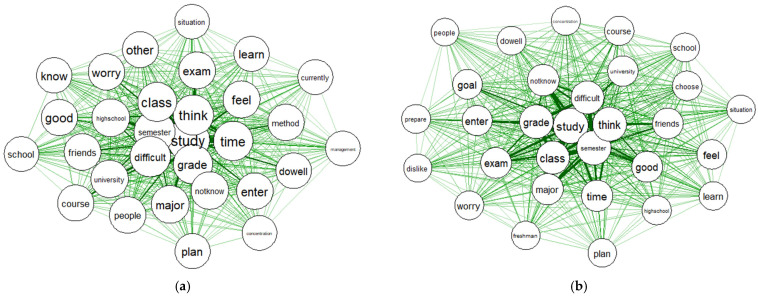
(**a**) Centrality of the top 30 keywords before COVID-19; (**b**) Centrality of the top 30 keywords after COVID-19.

Figure 4 presents the centrality of keywords identified from reports before and after COVID-19. The thicker the link, the higher degree of centrality.

### 3.3. Bigram

#### 3.3.1. Affiliated Department Tracks

A bigram illustrates a relationship between words. For the HSS track, the word “study” appeared with “hard’, “method”, “time”, “concentration”, and “university”. For the STEM track, it was frequently used with “hard”, “HighSchool”, “time”, “habit”, and “difficult”. The word “time” most frequently appeared with “management” in both tracks. “Semester” was also mentioned often, and it coappeared with “freshman”, “AcademicProbation”, “grade”, and “study” in the HSS track, and with “method”, “freshman”, “grade”, and “study” in the STEM track. The word “university” appeared with “study” in the HSS track and with “enter” in the STEM track; the word “exam” appeared with “study” in the HSS track but with “period” and “prepare” in the STEM track. Examining the top 20 ranks of the bigrams, the word relations of “GraduateSchool—enter”, “grade—low”, “procrastinate—habit”, and “exam—study” were identified only in the HSS track, while relations between “other—people”, “university—enter”, “club—activity”, and “grade—raise” were identified only in STEM track. The top 20 most frequently-appearing word relations for the HSS and STEM tracks are presented in Table 5.

#### 3.3.2. Coaching Issues before and after COVID-19

The results of the bigram analysis of the top 20 word relations are presented in Table 6. For coaching issues addressed before the outbreak of the pandemic, the word “study” most often appeared with “method”, “hard”, “time”, HighSchool”, and “concentration”. After the outbreak of the pandemic, it most frequently appeared with “hard”, “NotKnow”, “semester”, “time”, and “major.” Before COVID-19, the word “semester” most frequently appeared with “freshman”, “grade”, “sophomore”, and “study”, while appearing with “freshman”, “academic probation”, and “study” after COVID-19. “The word “time” appeared with the word “management” in both categories. The relations between “other—people”, “club—activity” appeared before COVID-19, and relations between “assignment—submit”, “persistently—study”, “procrastinate—habit”, and “friends—around me” appeared in the top 20 rank after COVID-19.

## 4. Discussion

### 4.1. Findings and Implications

The current study examined academic coaching intake session reports accumulated at a Korean university from 2017 to 2021 to investigate keywords and their relations using text timing analysis. In order to provide meaningful baseline information for coaches, educators, and university administrators, comparisons were made first between HSS track and STEM track students, and then between coaching issues before and after COVID-19. The findings and implication of the study were as follows.

#### 4.1.1. Common Tendency toward Frequency, Centrality, and Bigram across Categories

A common tendency toward frequency and centrality of keywords, as well as bigrams, were found across all categories. Most of all, it was notable that the word “study” had a dominant appearance, with the highest frequency and the highest centrality, in all categories. This finding should be viewed in relation to the word “learn”, which did not appear in the top 20 rank in any category. Even though the words study and learn are closely related, there is a noteworthy difference. According to Oxford Learner’s Dictionaries, the word study is defined as “to spend time learning about a subject by reading, going to college, etc.; to examine a problem, situation, group, etc. in detail in order to analyze or understand it” [44] and learn is defined as “to gain knowledge or skill by studying, from experience, from being taught, etc.; to gradually change your attitudes about something so that you behave in a different way.” [45] As the definition suggests, studying is usually associated with formal education, where it allows one to “read, memorize facts, and attend school, etc.” [46] and to engage in the cognitive work of inputting and processing information [47]. On the other hand, learning is related to the process of knowing and doing, in which one becomes skillful or knowledgeable about something which may also affect one’s attitude and behavior [47].

It is true that university students attend a formal educational institution that requires studying the necessary course materials, but they also enter into adulthood; adult learners have been found to have different characteristics and motives for learning compared to children, being more autonomous and focused on making changes in their lives [48,49]. However, findings indicate that university students in Korea mainly talked about studying when they signed up for coaching services. Considering other frequently addressed words such as “grade”, “exam”, “class”, and “course” across the categories, the data could be interpreted to suggest that students in Korea study for exams to get good grades in their classes.

Bigrams also showed that “study—hard” was in the top 3 rank of the most frequently appearing word sequence in all the categories, and other links, such as “study—time” and “study—plan” also appeared in all the categories. This result may reflect the context of Korean education in which students in secondary school are drilled into pursuing high academic achievements by cramming information and getting the right answers in exams [50,51]. Such a prolonged approach to education could have influenced the students’ perception that even in university, they are merely students who have to study the given materials for an exam or for a good grade, rather than seeing themselves as learners autonomously learning to better themselves. It would be important for students to recognize themselves as self-directed learners so that they could extend their experiences in university rather than focusing on studying for an evaluation.

Another notable finding was the common appearance of “HighSchool”, “university”, and “semester” in the top 20 rank of frequency as well as their centrality in all the categories. Bigrams also showed “university—enter” and “freshman—semester” sequences across the categories, as well as the “HighSchool—study” sequence, appearing in both tracks and before COVID-19. The challenges and changes students encounter when transitioning from high school to university have been addressed by previous research [24,25,26]. For students in Korea, high school years mainly focus on preparing for the university entrance exam [52], without deep consideration for the choice of their major or career path [53]. In high school, students had a clear goal—to enter a prestigious university—and knew how and what to study. Entering a four-year university in Seoul, Korea indicated that the students were high achieving students who had received a fairly high grade on the entrance exam. However, after entering a university, they have to compete with other well-performing students and adapt to a different learning environment while also planning for the future. Compared to their past, with high school years as their frame of reference, they may feel that they are not performing sufficiently, leading them to seek coaching. Thus, preparing students for the transition and guiding them to set appropriate future goals rather than referencing to their past success could help students with their campus life.

#### 4.1.2. Similarities and Differences between HSS Track and STEM Track

The findings showed similarities and differences in the intake session reports of students from the HSS and STEM tracks. First, the word “friends” appeared in the top 20 frequency and centrality for both tracks. University is not just a place for learning; it is also for engaging in various interpersonal relationships. Bowman et al. [54] found that social connection and relationship satisfaction with college friends were closely related to the sense of belonging and well-being of students. It was noteworthy that students seeking coaching for academic performance and success addressed “friends” frequently. Although exploring the specific context would be beyond the scope of the current study, it could be suggested that relationships and social support are an important factor affecting the academic achievement of students.

Second, words such as “major” and “NotKnow” appeared in both tracks, but had higher frequency and centrality in the HSS track. Students in Korea do not have sufficient chances to explore suitable majors or career paths prior to attending university [53], so they may wonder about their fit to their major as the semesters progress. Consideration of their major seemed to occur more often in the HSS track. This result should be viewed in line with the appearance of “plan” and “worry”, as well as “GraduateSchool—enter” relation in the top 20 rank only in HSS track. The context of what HSS track students plan for, or what they worry about, was not provided in the findings, but it could be hypothesized that their concerns are related to their future career trajectory. There is a growing concern for students in the HSS track regarding their career trajectory due to the rapid advancement of technology and the changing labor market [55]; the frequency of these words may reflect such uncertainty.

Third, in STEM track, the word “people” showed higher frequency and centrality This may reflect the university setting and culture, in which undergraduate students in STEM are often assigned to work in a lab with professors and graduate school students. Thus, the relationship with people may be an important factor affecting their academic performance. Additionally, noting that the “other—people” relation was found in the bigram analysis of the STEM track, it could be interpreted that students in the STEM track may be paying more attention to others’ performance or perspectives since they have more opportunities to work collaboratively with others in a lab setting.

Lastly, the word “method” appeared to have high frequency, along with “prepare” and “concentration” in the top 20 rank, albeit only in the STEM track. In the bigram analysis, the word “prepare” appeared with “exam”. In STEM track, the curriculum flowcharts were usually fixed and most courses required prerequisite learning (e.g., mathematics, physics) as students advanced into higher levels in their coursework [56]. Students need to accumulate specific knowledge in order to advance in their fields, and many quizzes and exams are involved in the process of student evaluation. Students enrolled in STEM tracks tend to struggle more with GPA than non-STEM students [57]. The extracted keywords could be interpreted in relation to the learning context of STEM track. STEM students talked more about searching for methods to deal with their academic affairs, preparing for exams, and trying to enhance concentration for their studies.

In summary, understanding the similarities and differences of coaching issues for the two tracks may help provide varied support that meets the needs of students.

#### 4.1.3. Similarities and Differences between Coaching Issues before and after COVID-19

The current study also compared the keywords of coaching issues before and after COVID-19. First, while the words “friends” and “people” showed high frequency and centrality before COVID-19, only “friends” had high frequency and centrality after COVID-19. This may be due to the social quarantine measures implemented in early 2020, mandating the university courses to be taught online and restricting social contact. Although students had access to online platforms to participate in class and keep in touch with their friends, encounters with people in general were restricted. Lampe et al. [58] found that college students used social networking services such as Facebook primarily to extend pre-existing offline relationships rather than to initiate new ones. This indicates that there were less opportunities to meet new people due to social distancing policies, and that students seeking coaching after the outbreak of the pandemic talked mainly about their close friends.

Second, the word “NotKnow” appeared before and after COVID-19 but showed a higher rank of frequency and centrality after COVID-19. Additionally, the bigram analysis only showed a frequent appearance of the “study—NotKnow” link after COVID-19. Studies have shown that learning in online spaces can cause great uncertainty and confusion [59] and that reduced contact with other learners and instructors may lead to a drop in academic performance [60]. Increased reference to not knowing something could reflect the uncertainty students experienced when engaging in online courses during the pandemic.

Lastly, the word “plan” had higher frequency and centrality after COVID-19, and the word “goal” appeared in the top 20 rank of frequency and centrality only after COVID-19. This should also be viewed in association with “assignment—submit”, “persistently—study”, and “procrastinate—habit”, relations that only appeared after COVID-19. These results reflected the phenomenon of students’ taking courses online, requiring them to set their own goals and make specific plans on their own, to manage assignment submissions, and to study persistently without procrastinating. These results indicated that, in the post-pandemic era where online courses are prevalent, self-management intervention would be beneficial for students.

#### 4.1.4. Implications Based on the Findings

Taking all the results into account, the following implications can be proposed: first, it would be important to help university students in Korea understand their roles as adult learners, and not students passively taking in information. Based on theories and approaches of adult learning, such as andragogy [49], coaches could work with individual students to enhance their understanding of themselves, explore the meaning of learning, and empower them to take initiative for learning. Educators could design and implement various instructional strategies so that students could take part in their own learning process [48]. At a university level, university administrators could implement policies and systems to foster generic competencies of students, rather than evaluating them based primarily on grades, in order to help students grow into more autonomous and adaptive learners [3,4].

Second, coaches and educators should recognize the influence of social relationships on students’ academic performance and success and devise interventions or instructions that promote interpersonal skills, such as conflict resolution, communication, and a sense of belongingness. Specifically, with the prolonged pandemic, students’ campus life was moved to online classrooms, restricting the level of interaction among peers and with instructors. Coaches and educators should assess the needs of students and provide them with possible opportunities to interact with one another in class and in coaching sessions.

Third, ways to enhance self-management skills may be necessary for students adapting to online campus life. Martin [61] emphasized that educators needed to be mindful of delivery of instructions, managing the quality of contents, and motivating students in order to promote their self-regulation and management while engaging in online learning. Coaching could also help students with self-regulation, including time management and organization [13].

Finally, challenges that students encounter regarding the changing trajectory of their majoring field, especially for those in the HSS track, should be recognized. There have been studies examining the digital literacy of HSS students [62], and developing theories and models to converge STEM and HSS [63]. In alignment with such studies, university-level approaches providing opportunities for convergent majors should be implemented to better prepare students for the rapidly changing world.

### 4.2. Limitations and Directions for Future Research

The current study had certain limitations. First, the text mining approach used in the study was an effective way to extract information from a vast amount of text, but it was limited in that it could not provide in-depth understanding of the context in which the text was written, or identify specific variables and their relations. The current study investigated the keywords and their co-occurrences, providing only descriptive and exploratory understanding. Additional qualitative research will be needed to analyze the experiences of students comprehensively and to identify specific ways for coaches, educators, and university administrators to provide necessary student support.

Second, the current study made comparisons between students’ affiliated tracks and between issues before and after COVID-19, which were prominent topics of interest for Korean universities. However, as the aforementioned literature review shows, there were various factors affecting students’ academic life, and students’ characteristics, such as gender and grade level, could also affect their academic success. Hence, future study will be needed to examine factors other than affiliated tracks and the impact of the pandemic in order to comprehensively understand students’ academic challenges and gain insight about necessary interventions.

Third, the findings reflected the educational culture and situation in Korea, but university life and academic challenges students face may differ in other cultural settings. Thus, further study will be needed to compare coaching issues addressed in different university settings in various countries.

Fourth, the study only investigated the initial intake session reports, in which students described their current academic concerns and perceived barriers. However, as coaching proceeds and students gain understanding of themselves and their situations, they could discover other issues that were affecting their academic performance, or their perception of the problem could change. In order to enhance understanding of students’ academic concerns and barriers and to devise better coaching interventions, future studies must investigate the process and outcome of academic coaching.

Despite these limitations, the current study was meaningful in that it examined accumulated authentic data collected from students who were voluntarily seeking coaching services. The findings provided insight into the needs of students and helped to elucidate directions for individualized student support, to be provided at the university level to promote academic success.

## 5. Conclusions

The current study examined the intake session reports of an academic coaching program provided at a university in Seoul, Korea, using text mining analysis. The study examined the frequency and centrality of words and their relations based on students’ affiliated department tracks and coaching issues before and after the pandemic. The results of the study provided baseline information from the authentic and natural dataset to inform coaches, educators, and policymakers as they work to devise appropriate interventions to satisfy the needs of the students. Although the current study was limited in its ability to explain the specific context of students’ academic concerns, the results could provide insight into understanding the issues that students bring into their academic coaching sessions. Based on the results of the current study, further study could provide additional evidence to aid in the enhancement of academic coaching for university students.

## Figures and Tables

**Table 1:** Frequencies of the top 20 keywords for HSS and STEM tracks.

Rank	HSS (*n* = 210)		STEM (*n* = 171)	
	Word	Frequency	Word	Frequency
1	Study	1002	Study	888
2	Semester	530	Semester	411
3	Think	419	Grade	354
4	Grade	364	Think	326
5	Difficult	323	Difficult	306
6	Time	321	Class	289
7	Class	316	Time	270
8	Major	233	Exam	190
9	Friends	224	Friends	185
10	NotKnow	212	Feel	174
11	Feel	198	HighSchool	171
12	Exam	193	Major	156
13	HighSchool	184	Now	15
14	Now	173	University	149
15	Course	168	NotKnow	145
16	University	168	Method	140
17	Plan	161	Course	136
18	School	153	Prepare	136
19	Enter	146	People	135
20	Worry	143	Concentration	123

**Table 2:** Frequencies of the top 20 keywords before and after COVID-19.

Rank	before COVID-19 (*n* = 256)		after COVID-19 (*n* = 127)	
	Word	Frequency	Word	Frequency
1	Study	1263	Study	627
2	Semester	677	Semester	264
3	Think	519	Grade	227
4	Grade	491	Think	226
5	Difficult	467	Class	202
6	Time	436	Difficult	162
7	Class	403	Time	155
8	Friends	309	Major	131
9	Feel	288	NotKnow	130
10	Exam	269	Exam	114
11	HighSchool	269	Friends	100
12	Major	258	Course	93
13	Now	247	University	93
14	NotKnow	227	Plan	92
15	University	224	Goal	87
16	Course	211	Enter	86
17	Method	208	HighSchool	86
18	People	207	Concentration	84
19	Worry	191	Feel	84
20	Plan	184	Good	81

**Table 3:** Centrality of the top-ranked words for HSS and STEM tracks.

Rank	HSS (*n* = 210)		STEM (*n* = 171)	
	Word	Centrality	Word	Centrality
1	study	0.689	study	0.660
2	think	0.503	think	0.437
3	semester	0.450	semester	0.391
4	class	0.378	class	0.377
5	difficult	0.374	difficult	0.376
6	grade	0.341	grade	0.360
7	time	0.325	time	0.325
8	NotKnow	0.325	feel	0.281
9	friends	0.291	friends	0.265
10	major	0.276	now	0.248
11	feel	0.272	HighSchool	0.246
12	now	0.271	NotKnow	0.239
13	HighSchool	0.238	exam	0.238
14	worry	0.238	major	0.227
15	exam	0.230	university	0.220
16	plan	0.230	professor	0.212
17	university	0.226	people	0.210
18	course	0.224	course	0.208
19	school	0.220	situation	0.208
20	learn	0.201	prepare	0.206

**Table 4:** Centrality of the top-ranked words before and after COVID-19.

Rank	before COVID-19 (*n* = 256)		after COVID-19 (*n* = 127)	
	Word	Centrality	Word	Centrality
1	study	0.698	study	0.624
2	think	0.521	think	0.400
3	semester	0.483	semester	0.345
4	difficult	0.443	class	0.338
5	class	0.397	grade	0.314
6	grade	0.376	difficult	0.270
7	time	0.370	NotKnow	0.261
8	feel	0.341	time	0.249
9	friends	0.322	major	0.207
10	now	0.321	friends	0.193
11	HighSchool	0.291	feel	0.184
12	NotKnow	0.287	exam	0.176
13	exam	0.278	now	0.176
14	major	0.275	course	0.173
15	university	0.262	HighSchool	0.168
16	worry	0.262	plan	0.164
17	people	0.260	worry	0.164
18	professor	0.251	goal	0.159
19	situation	0.248	university	0.158
20	know	0.247	school	0.155

**Table 5:** Bigram of word relations for HSS and STEM tracks.

Rank	HSS (*n* = 210)			STEM (*n* = 171)		
	Word1	Word2	Frequency	Word1	Word2	Frequency
1	Study	Hard	65	Study	Hard	65
2	Time	Management	56	Semester	Method	58
3	Study	Study	47	Time	Management	53
4	Study	Method	44	Study	Study	40
5	Study	Time	39	Freshman	Semester	38
6	Semester	Semester	35	Exam	Period	34
7	University	Enter	35	HighSchool	Study	32
8	GraduateSchool	Enter	33	Semester	Grade	31
9	Freshman	Semester	31	Study	Time	31
10	Study	Concentration	29	Other	People	30
11	University	Study	29	University	Enter	26
12	HighSchool	Study	28	Semester	Semester	25
13	Semester	AcademicProbation	28	Study	Habit	25
14	Semester	Grade	28	Exam	Prepare	23
15	Semester	Study	28	Study	Difficult	23
16	Grade	Low	27	Study	Plan	23
17	Hours	Study	27	Club	Activity	22
18	Procrastinate	Habit	27	Grade	Grade	22
19	Study	Plan	27	Grade	Raise	22
20	Exam	Study	26	Semester	Study	22

**Table 6:** Bigram of word relations before and after COVID-19.

Rank	before COVID-19 (*n* = 256)			after COVID-19 (*n* = 127)		
	Word1	Word2	Frequency	Word1	Word2	Frequency
1	Study	Method	88	Study	Hard	52
2	Time	Management	81	Study	Study	28
3	Study	Hard	78	Time	Management	28
4	Study	Study	57	University	Enter	25
5	Study	Time	52	GraduateSchool	Enter	22
6	Freshman	Semester	50	Grade	Raise	20
7	Semester	Grade	49	Study	NotKnow	20
8	Semester	Semester	49	Exam	Prepare	19
9	HighSchool	Study	45	Freshman	Semester	19
10	Other	People	43	Assignment	Submit	18
11	Club	Activity	36	Semester	AcademicProbation	18
12	University	Enter	36	Semester	Study	18
13	Exam	Period	35	Study	Time	18
14	Sophomore	Semester	34	Grade	Good	17
15	Study	Concentration	34	Major	Study	16
16	Study	Plan	34	Persistently	Study	16
17	Grade	Low	33	Procrastinate	Habit	16
18	GraduateSchool	Enter	32	Study	Plan	16
19	Semester	Study	32	AroundMe	Friends	15
20	University	Study	32	Exam	Study	15

## Data Availability

The data presented in this study are available upon request from the corresponding author. The data are not publicly available due to the bylaws of the institution.

## Appendix A

**Table A1:** Keywords in HSS track with frequency of 30 or more.

Rank	Word	Frequency	Word	Frequency	Word	Frequency	Word	Frequency
1~4	study	1002	semester	530	think	419	grade	364
5~8	difficult	323	time	321	class	316	major	233
9~12	friends	224	NotKnow	212	feel	198	exam	193
13~16	HighSchool	184	now	173	course	168	university	168
17~20	plan	161	school	153	enter	146	worry	143
21~24	good	142	concentration	137	Korean	128	learn	128
25~28	people	120	DoWell	118	graduate	116	GraduateSchool	116
29~32	prepare	116	assignment	115	contents	112	interest	112
33~36	method	111	understand	111	know	109	other	107
37~40	situation	107	student	105	choose	104	hours	103
41~44	start	103	freshman	101	professor	100	management	99
45~48	career	97	procrastinate	96	goal	94	English	91
49~52	problem	91	dislike	90	JobHunting	89	continuously	87
53~56	lack	77	task	77	currently	76	parents	76
57~60	LeaveOfAbsence	74	AcademicProbation	71	stress	71	work	71
61~64	interesting	70	Korea	70	like	70	life	69
65~68	MajorCourse	68	reason	68	habit	67	hard	65
69~72	activity	64	sophomore	64	DoubleMajor	63	GiveUp	63
73~76	anxious	60	apply	59	department	59	AroundMe	58
77~80	lose	58	read	58	effort	57	club	56
81~84	submit	55	lecture	54	motivation	53	relationship	53
85~88	sleep	52	well	52	ability	51	military	51
89~92	efficient	50	ReturnToSchool	50	suitable	50	want	50
93~96	help	49	mother	49	family	48	talk	48
97~100	studies	47	ByMyself	46	math	46	need	46
101~104	UntilNow	46	ElectiveCourse	45	different	44	find	44
105~108	presentation	44	WorkHard	44	cellphone	43	home	43
109~112	score	43	vacation	43	alone	42	current	42
113~116	feeling	42	low	42	memorization	42	rest	42
117~120	review	42	change	41	credit	41	difficulty	41
121~124	confident	40	experience	40	improve	39	use	38
125~128	decide	37	inquire	37	follow	36	learning	36
129~132	midterm	36	PartTimeJob	36	RetakingCollegeEntranceExam	36	execute	35
133~136	academic	34	adapt	34	CampusLife	33	China	33
137~140	game	33	high	33	TeamProject	33	write	33
141~144	writing	33	burden	32	persistently	32	questions	32
145~148	undergraduate	32	deadline	31	organize	31	research	31
149~152	avoid	30	Chinese	30	computer	30	cramming	30
153~156	doctoral	30	period	30	physics	30	process	30

**Table A2:** Keywords in STEM track with frequency of 30 or more.

Rank	Word	Frequency	Word	Frequency	Word	Frequency	Word	Frequency
1~4	study	888	semester	411	grade	354	think	326
5~8	difficult	306	class	289	time	270	exam	190
9~12	friends	185	feel	174	HighSchool	171	major	156
13~16	now	153	university	149	NotKnow	145	method	140
17~20	course	136	prepare	136	people	135	concentration	123
21~24	enter	122	worry	120	professor	117	DoWell	115
25~28	plan	115	good	114	situation	108	management	105
29~32	assignment	103	freshman	100	learn	100	English	99
33~36	interest	99	other	99	contents	95	stress	95
37~40	GraduateSchool	93	career	85	choose	84	goal	84
41~44	know	82	school	81	graudate	79	Korean	77
45~48	military	76	LeaveOfAbsence	74	like	74	relationship	74
49~52	JobHunting	69	dislike	68	task	68	lack	67
53~56	hard	66	activity	65	motivation	65	understand	65
57~60	habit	64	problem	63	work	63	life	62
61~64	club	60	currently	60	need	60	talk	60
65~68	MajorCourse	59	start	58	continuously	57	period	57
69~72	WorkHard	57	student	55	apply	54	different	54
73~76	submit	54	procrastinate	53	want	53	ReturnToSchool	51
77~80	effort	50	read	50	GiveUp	48	help	48
81~84	sophomore	48	vacation	48	well	48	alone	47
85~88	anxious	47	department	47	thesis	46	current	45
89~92	doctoral	45	find	45	Korea	45	AcademicProbation	44
93~96	hours	44	studies	44	article	43	experience	43
97~100	review	43	peers	42	difficulty	41	efficient	41
101~104	lecture	41	undergraduate	41	DoubleMajor	40	research	40
105~108	feeling	39	suitable	39	reason	38	write	38
109~112	AroundMe	37	lose	37	writing	37	burden	36
113~116	home	36	persistently	36	questions	36	sleep	36
117~120	cellphone	35	important	35	math	35	parents	35
121~124	ability	34	follow	33	MiddleSchool	33	presentation	33
125~128	academic	32	decide	32	diligently	32	inquire	32
129~132	lab	32	learning	32	low	32	master	32
133~136	UntilNow	32	memorization	31	midterm	31	myself	31
137~140	PrivateInstitute	31	process	31	program	31	rest	31
141~143	ByMyself	30	finish	30	personality	30		

**Table A3:** Keywords before COVID-19 (2017–2019) with frequency of 30 or more.

Rank	Word	Frequency	Word	Frequency	Word	Frequency	Word	Frequency
1~4	study	1263	semester	677	think	519	grade	491
5~8	difficult	467	time	436	class	403	friends	309
9~12	feel	288	exam	269	HighSchool	269	major	258
13~16	now	247	NotKnow	227	university	224	course	211
17~20	method	208	people	207	worry	191	plan	184
21~24	enter	182	concentration	176	good	175	prepare	175
25~28	DoWell	171	Korean	171	professor	171	school	167
29~32	situation	161	GraduateSchool	158	other	158	know	155
33~36	learn	151	interest	148	contents	147	freshman	147
37~40	management	147	English	142	assignment	139	graduate	139
41~44	student	134	career	125	understand	125	stress	122
45~48	choose	121	problem	116	start	115	currently	110
49~52	hours	108	relationship	108	Korea	105	like	105
53~56	lack	104	continuously	103	military	103	JobHunting	101
57~60	LeaveOfAbsence	100	work	100	task	99	club	96
61~64	MajorCourse	96	goal	91	dislike	88	activity	86
65~68	anxious	86	sophomore	86	talk	86	habit	85
69~72	department	84	life	84	read	83	need	82
73~76	procrastinate	81	parents	80	hard	79	help	79
77~80	different	78	reason	78	apply	77	interesting	75
81~84	DoubleMajor	73	thesis	72	submit	70	want	69
85~88	motivation	67	well	67	WorkHard	67	difficulty	66
89~92	AcademicProbation	65	alone	65	cellphone	65	doctoral	65
93~96	ability	64	efficient	64	AroundMe	63	home	63
97~100	undergraduate	63	article	62	current	62	vacation	62
101~104	ReturnToSchool	61	decide	60	lose	60	low	60
105~108	sleep	60	find	59	GiveUp	59	mother	59
109~112	research	59	ElectiveCourse	58	period	58	review	58
113~116	studies	58	math	57	peers	57	ByMyself	56
117~120	experience	55	feeling	55	follow	55	presentation	55
121~124	score	55	memorization	54	suitable	54	writing	54
125~128	effort	52	MiddleSchool	52	use	52	inquire	51
129~132	master	51	lab	51	write	50	RetakingCollegeEntranceExam	50
133~136	burden	49	learning	49	lecture	49	process	49
137~140	PartTimeJob	46	Chinese	45	PrivateInstitute	45	academic	44
141~144	change	44	credit	44	finish	44	job	44
145~148	China	43	confident	43	family	43	midterm	43
149~152	program	43	game	42	improve	42	information	41
153~156	organize	41	questions	41	rest	41	upperclassman	41
157~160	important	40	myself	40	StudyingAbroad	40	UntilNow	40
161~164	book	39	check	39	counseling	39	execute	39
165~168	junior	39	beginning	38	deadline	38	materials	38
169~172	personality	38	result	38	CollegeEntranceExam	37	InternationalStudent	37
173~176	persistently	37	satisfied	37	bad	36	CampusLife	36
177~180	field	36	language	35	discharged	34	father	34
181~184	high	34	adapt	33	Exchangestudent	33	teacher	33
185~188	atmosphere	32	avoid	32	notetaking	32	physics	32
189~192	schedule	32	transfer	32	answer	31	computer	31
193~196	EMI	31	money	31	participate	31	report	31
197~200	diligently	30	easy	30	quit	30	TeamProject	30

**Table A4:** Keywords after COVID-19 (2020–2021) with frequency of 30 or more.

Rank	Word	Frequency	Word	Frequency	Word	Frequency	Word	Frequency
1~4	study	627	semester	264	grade	227	think	226
5~8	class	202	difficult	162	time	155	major	131
9~12	NotKnow	130	exam	114	friends	100	course	93
13~16	university	93	plan	92	goal	87	enter	86
17~20	HighSchool	86	concentration	84	feel	84	good	81
21~24	assignment	79	now	79	learn	77	prepare	77
25~28	worry	72	dislike	70	procrastinate	68	choose	67
29~32	school	67	interest	63	DoWell	62	contents	60
33~36	career	57	JobHunting	57	management	57	graduate	56
37~40	effort	55	freshman	54	situation	54	GiveUp	52
41~44	hard	52	GraduateSchool	51	motivation	51	understand	51
45~48	AcademicProbation	50	English	48	LeaveOfAbsence	48	other	48
49~52	people	48	life	47	habit	46	lecture	46
53~56	professor	46	start	46	task	46	stress	44
57~60	activity	43	method	43	continuously	41	lack	40
61~64	ReturnToSchool	40	hours	39	like	39	submit	39
65~68	problem	38	UntilNow	38	apply	36	know	36
69~72	lose	35	suitable	35	Korean	34	want	34
73~76	work	34	WorkHard	34	studies	33	well	33
77~80	AroundMe	32	rest	32	MajorCourse	31	parents	31
81~84	persistently	31	diligently	30	DoubleMajor	30	find	30
